# A Real-Time PCR Assay for Bat SARS-Like Coronavirus Detection and Its Application to Italian Greater Horseshoe Bat Faecal Sample Surveys

**DOI:** 10.1100/2012/989514

**Published:** 2011-11-22

**Authors:** Andrea Balboni, Laura Gallina, Alessandra Palladini, Santino Prosperi, Mara Battilani

**Affiliations:** ^1^Dipartimento di Scienze Mediche Veterinarie, Alma Mater Studiorum-Università di Bologna, Via Tolara di Sopra 50, 40064 Ozzano Emilia, Italy; ^2^Dipartimento di Biologia Animale, Università degli Studi di Pavia, Via Taramelli 24, 27100 Pavia, Italy

## Abstract

Bats are source of coronaviruses closely related to the severe acute respiratory syndrome (SARS) virus. Numerous studies have been carried out to identify new bat viruses related to SARS-coronavirus (bat-SARS-like CoVs) using a reverse-transcribed-polymerase chain reaction assay. However, a qualitative PCR could underestimate the prevalence of infection, affecting the epidemiological evaluation of bats in viral ecology. In this work an SYBR Green-real time PCR assay was developed for diagnosing infection with SARS-related coronaviruses from bat guano and was applied as screening tool in a survey carried out on 45 greater horseshoe bats (*Rhinolophus ferrumequinum*) sampled in Italy in 2009. The assay showed high sensitivity and reproducibility. Its application on bats screening resulted in a prevalence of 42%. This method could be suitable as screening tool in epidemiological surveys about the presence of bat-SARS-like CoVs, consequently to obtain a more realistic scenario of the viral prevalence in the population.

## 1. Introduction

Among the human viral epidemics which have emerged in the past ten years, one of the most important is represented by the outbreak of severe acute respiratory syndrome (SARS) which appeared in China in 2002-2003, rapidly causing a human epidemic [1].

Several studies showed, firstly, that a new coronavirus was the aetiological agent of SARS, the SARS-CoV [2], and subsequently bats were the natural reservoir for several viruses closely related genetically to the SARS-CoV, the SARS-like coronaviruses (SARS-like CoVs) [3]. At present, the presence of SARS-like CoVs in bats has been demonstrated in Asia, Africa, and Europe [4–9], showing the wide diffusion of these viruses.

Bats play a critical role in the ecology and evolution of these viruses; therefore, numerous studies have been carried out to identify new bat-SARS-like CoVs and to understand the mechanisms of transmission from animal hosts to humans.

In most of the surveys carried out on bat-SARS-like CoVs, the preferred technique for the initial screening of population samples has been a reverse-transcribed-polymerase chain reaction (RT-PCR) assay or an RT-nested PCR assay on RNA extracted from faecal samples. The primers used for the PCR reaction generally amplified a fragment of the RNA-dependent RNA polymerase gene (RdRp, the 12th nonstructural protein encoded by ORF1a, b), a conserved feature of the coronavirus genome which was also frequently used for subsequent phylogenetic analysis [4, 8, 10–16]. However, this technique may not have an optimal sensitivity, causing an underestimation of the true prevalence of infection, which may be due to a low amount of viral clearance in the faeces during the infection, too low to be detected by traditional PCR methods.

Real-time PCR assay is a method with a significantly lower limit of detection than conventional PCR, allowing to increase the sensitivity of diagnostic test. In spite of high sensitivity, until now, only a few authors have used this technique in their epidemiological surveys [5, 10, 13]. The developed real-time PCRs described by other authors have used primers designed on viral sequences found in local geographical areas; therefore, it is uncertain whether these assays are able to detect viruses from other areas, in light of the high genetic variability of the coronaviruses.

The aim of this work was to develop a real-time PCR assay for diagnosing infection with SARS-related coronaviruses from bat guano in order to use it as a screening tool in epidemiological surveys for the detection of the viruses. For this purpose, an SYBR Green real-time PCR assay, amplifying a fragment of the RdRp gene for the generic detection of the coronavirus [17], was modified to increase the specificity towards the SARS-related coronavirus. The developed SYBR Green real-time PCR techniques were applied to an SARS-like coronavirus survey carried out on 45 greater horseshoe bats (*Rhinolophus ferrumequinum*) which were sampled in Italy in 2009, resulting in a prevalence of coronavirus infection of 42%.

## 2. Materials and Methods

### 2.1. Sampling from Bats

**Figure 1:**
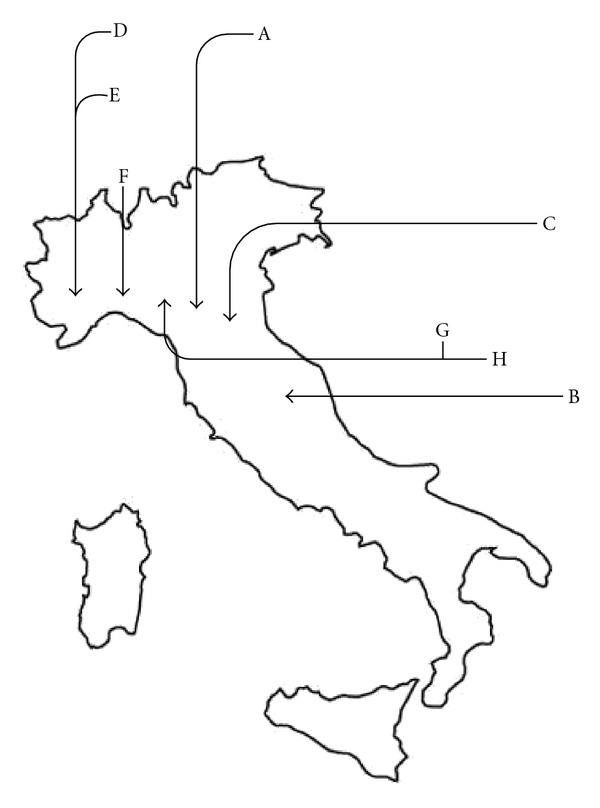
Distribution of bat sampling in Italy. Sites of bat sampling from August to November 2009. For correspondence between letters and places, see Table 1.

Forty-five greater horseshoe bat (*Rhinolophus ferrumequinum*) specimens were captured from different roosts in all of Italy, including caves, mines, and abandoned houses, over a 4-month period (from August to November 2009) (Figure 1 and Table 1). All captures were authorised by the Italian Ministry of the Environment and were part of a Ph.D. project on *Rhinolophus ferrumequinum* conservation.

The bats were caught using harp traps and placed individually into cotton bags before subsequent investigation started. Once species, sex, age category (juvenile, subadult, adult), forearm length, and weight were determined, the bats were released at their capture site.

The faeces were immediately collected when bats produced fresh bolus during handling and kept in 2 mL of RNAlater RNA stabilisation reagent (QIAGEN, Hilden, Germany) permitting RNA conservation during transport. The samples were then conserved at −80°C before processing.

### 2.2. RNA Extraction and cDNA Synthesis

Faecal boluses of each sampled bat were suspended in 200 *μ*L of phosphate buffered saline (PBS) pH 7.2 ± 0,2, and viral RNA extraction was performed beginning with 140 *μ*L of suspended faeces by using the QIAamp Viral RNA (Qiagen, Hilden, Germany) following the supplier's recommendations. Extracted RNA was stored at −80°C. First-strand cDNA was synthesised using random hexamers with the ImProm-II Reverse Transcription System (Promega Corporation, Madison, WI, USA), according to the manufacturer's instructions, and was stored at −20°C. 

### 2.3. Primer Design

**Figure 2:**
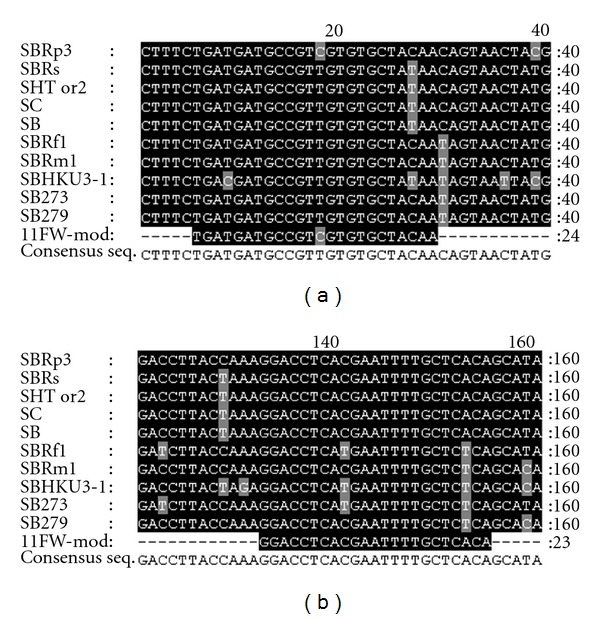
Multiple sequence alignment of primer binding sites. The alignments include 10 SARS-related coronavirus reference strains: SBRp3 (Bat SARS CoV Rp3/2004, DQ071615, identified from *Rhinolophus pearsoni*), SBRs (SARS coronavirus Rs_672/2006, FJ588686, identified from *Rhinolophus sinicus*), SHTor2 (SARS coronavirus Tor2, AY274119, identified from Human), SC (Civet SARS CoV SZ16/2003, AY304488, identified from* Paguma larvata*), SB (SARS coronavirus isolate CFB/SZ/94/03, AY545919, identified from* Melogale moschata*), SBRf1 (Bat SARS CoV Rf1/2004, DQ412042, identified from *Rhinolophus ferrumequinum*), SBRm1 (Bat SARS CoV Rm1/2004, DQ412043, identified from *Rhinolophus macrotis*), SBHKU3-1 (Bat coronavirus HKU3, DQ022305, identified from *Rhinolophus sinicus*), SB273 (Bat CoV 273/2005, DQ648856, identified from *Rhinolophus ferrumequinum*), and SB279 (Bat CoV 279/2005, DQ648857, identified from *Rhinolophus macrotis*).

The forward and reverse primers were designed on the basis of the 11FW and 13RV degenerate primers which amplified a fragment of RNA-dependent RNA polymerase gene (RdRp), previously published by Escutenaire et al. [17]. The sequences of the two primers were modified on the basis of a multiple sequence alignment including 10 SARS-related coronavirus reference strains available from the GenBank database in order to increase the specificity of the primers towards the bat-SARS-like coronaviruses (Figure 2). Reference sequences were aligned using ClustalW software implemented with BioEdit version 7.0.5.

Using 11FW-modified (5′-TGA TGA TGC CGT CGT GTG CTA CAA-3′) and 13RV-modified (5′- TGT GAG CAA AAT TCG TGA GGT CC-3′) primers, a 168 bp fragment located between the nt 15647–15814 (Coronavirus, SARS-CoV Tor2; NC_004718) was able to be detected.

### 2.4. Preparation of Standard DNA

A pCR4 plasmid (Invitrogen, Carlsbad, California, USA) containing a copy of the RdRp target sequence was produced as the external standard for the construction of the assay standard curve.

The template DNA to be inserted as a target sequence in the plasmid vector was produced by amplifying the cDNA obtained from sample 893/09-11, an Italian bat-SARS-like CoV detected and only partially sequenced by the authors in a preceding survey [4]. A conventional PCR was carried out by Taq DNA Polymerase (Qiagen, Hilden, Germany) using 11FW-modified and 13RV-modified primers; the thermal cycler conditions consisted of an initial incubation at 94°C for 5 min followed by 50 cycles of denaturation at 94°C for 40 sec, annealing at 50°C for 40 sec, and polymerisation at 72°C for 40 sec. A final cycle of extension at 72°C for 30 min was performed to produce a single 3′ deoxiadenosine (A) overhang. 

The amplification product was purified by the High Pure PCR Product Purification Kit (Roche Diagnostics, Mannheim, Germany) following the supplier's recommendations and was cloned into the pCR4 vector using the TOPO TA Cloning Kit (Invitrogen, Leek, The Netherland), according to the manufacturer's instructions. The resulting recombinant plasmid purification was carried out using the PureLink Quick Plasmid Miniprep Kit (Invitrogen, Leek, the Netherland); purity was assessed by a spectrophotometer using the 260/280 nm ratio. 

In order to simulate the amplification efficiency of viral reverse-transcribed RNA more closely and to avoid the presence of supercoiled plasmid DNA, the plasmid was linearised above the bat-SARS-like CoV fragment sequence using restriction endonuclease Spe I (Fermentas, Burlington, Ontario, Canada). The linearised plasmid was visualised and quantified by electrophoresis on 1% (w/v) agarose gel stained with ethidium bromide in 1x standard TAE buffer and using a Quick-Load 1Kb DNA Ladder (New England BioLabs, Ipswich, MA, USA). The copy number (CN) of standard plasmid DNA was calculated using the equation described by the US Environmental Protection Agency protocol [18].

### 2.5. SYBR Green Real-Time PCR

The real-time PCR was performed using the SYBR Premix Ex Taq II (Takara Bio inc., Shiga, Japan) and the Rotor-Geen 3000 system (Corbett Research, Mortlake, NSW, Australia). The fluorescence signal was acquired on the FAM channel (multi channel machine, source, 470 nm; detector, 510 nm; gain set to 5) with a fluorescence reading taken at the end of each elongation step.

The SYBR Green real-time PCR was performed in a final volume of 25 *μ*L containing 12.5 *μ*L of SYBR Premix Ex Taq II, 0.4 *μ*M of forward (11FW-modified) and reverse (13RV-modified) primers and 2 *μ*L of template DNA. Autoclaved nanopure water was added to arrive at a final volume of 25 *μ*L.

Each run consisted of an initial incubation for activation of the hot-start DNA polymerase at 95°C for 30 sec followed by 40 cycles of denaturation at 95°C for 10 sec, annealing at 55°C for 20 sec, and polymerization at 72°C for 20 sec. During the melt cycle, the temperature was increased by increments of 1°C from 72°C to 95°C.

Specimens were considered positive if the fluorescence curve in the amplification plot showed an exponential increase, and a specific melting peak was observed.

### 2.6. Standard Curve and Limit of Detection (LOD)

The SYBR Green real-time PCR standard curve was generated by serial 10-fold dilutions of recombinant plasmid with a known copy number (from 1 × 10^8^ to 1 × 10^−1^ copies/*μ*L). These dilutions were tested in triplicate and used as quantification standards to construct the standard curve by plotting the plasmid copy number against the corresponding threshold cycle values (Ct). The threshold was determined using the Autofind Threshold function of the Rotor-Gene 3000, which scans the range of the threshold levels to obtain the best fit of the standard curve through the samples which have been defined as standards. The limit of detection (LOD) of the reaction was determined based on the highest dilution of plasmid possible to amplify with good reproducibility.

To verify the specificity of the reaction, a melting curve analysis and electrophoresis on agarose gel were carried out for the products of the SYBR Green real-time PCR reaction. Five microliters of the amplicons were electrophoresed in 2% (w/v) agarose gel stained with ethidium bromide in 1x standard tris-acetate-EDTA (TAE) buffer and visualised by ultravioltet (UV) light; MassRuler Low-Range DNA Ladder (Fermentas, Burlington, Ontario, Canada) was used to check that amplicons of the expected size were present.

### 2.7. Diagnostic Sensitivity and Specificity

The analytical sensitivity and efficiency of real-time PCR is reflected by the LOD, which was assessed as described previously.

The specificity of the assay was evaluated using several dilutions of the recombinant plasmid together with a bat-SARS-like CoV, coronaviruses of different genera, and other RNA and DNA viruses, in triplicate.

The cDNA obtained from RNA extracted from faecal sample 893/09-11 [4] was used as a standard to test the amplification of the bat-SARS-like CoV while coronaviruses of other genera tested were feline coronavirus FCoV (Alphacoronavirus, strain 420, cDNA obtained from RNA extracted from lymph node, [19]), and infectious bronchitis virus IBV (Gammacoronaviru**s,** strain M41, DQ834384, cDNA obtained from RNA extracted from infected cells, kindly provided by Professor Elena Catelli). To further determine the specificity of the amplification reaction, the canine distemper virus CDV (Morbillivirus, Onderstepoort strain, NOBIVAC PUPPY CP, Nobivac, Boxmeer, The Netherlands, cDNA obtained from RNA extracted from vaccine suspension) and the feline panleukopenia virus FPV (Parvovirus, strain 1033/09, DNA extracted from tongue, [20]) were tested as noncoronavirus RNA viruses and as DNA viruses, respectively. A melting curve analysis of the amplification products obtained was carried out.

### 2.8. Intra- and Interassay Variability

In order to determine the intraassay variability of the technique for the standard plasmid and for samples, seven successive 10-fold dilutions (from 2 × 10^6^ to 2 × 10^0^ copies/*μ*L) of recombinant plasmid were tested in triplicate within the same run, and the same was done for three samples with different viral concentrations (the highest viral concentration detected among the sampled bats (B1): 10^2^, and two low viral concentrations near the limit of detection (B2 and B3): 10^0^) (Table 2).

The interassay variability was tested on bat samples by testing the three samples with different viral concentrations (B1, B2, and B3) in triplicate on three different days (Table 2).

Mean, standard deviation (SD), and coefficient of variation (CV) were calculated. In particular, the CV was calculated as the percentage of the ratio of standard deviation and the mean of the copies obtained, in accordance with the US Environmental Protection Agency protocol [18].

### 2.9. Detection of Coronavirus in Bat Faecal Samples Using SYBR Green Real-Time PCR

After optimisation of the SYBR Green real-time PCR assay, for each run, duplicates of six 10-fold dilutions of the standard plasmid, triplicates of the viral reverse-transcribed RNA of the bat samples, and a no template control were simultaneously subjected to analysis.

Bat samples were considered positive if the mean of three replicates was greater than the LOD.

To confirm the expected size of the reaction products, five microliters of the amplicons were electrophoresed in 2% (w/v) agarose gel stained with ethidium bromide in 1x standard tris-acetate-EDTA (TAE) buffer and visualised by ultravioltet (UV) light; MassRuler Low-Range DNA Ladder (Fermentas, Burlington, Ontario, Canada) was used to check the size of DNA fragments.

## 3. Results

### 3.1. Development and Validation of SYBR Green Real-Time PCR

The linearity and efficiency of the SYBR Green real-time PCR were determined by generating a standard curve in which serial 10-fold dilutions of recombinant plasmid were tested. The standard curve was generated by plotting the real-time PCR threshold cycle numbers (Ct) of each dilution against the known copy numbers of recombinant plasmid. The resulting slope showed a linear relationship over 9 orders of magnitude, ranging from approximately 1 × 10^0^ to 1 × 10^8^ copies/*μ*L. The slope was −3.36 with a coefficient of determination (*R* ^2^) > 0.99 and a reaction efficiency (*E*) of 0.99, calculated from the slope (*S*) using *E* = 10^(−1/*S*)^ − 1.

**Figure 3:**
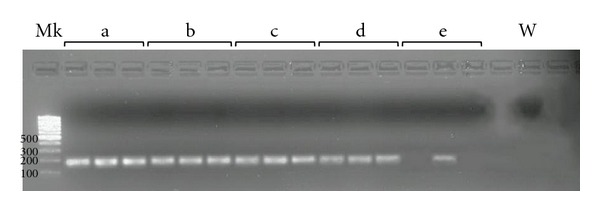
Real-time PCR reaction products checked on agarose gel stained with ethidium bromide in standard tris-acetate-EDTA (TAE) buffer: serial 10-fold dilutions of recombinant plasmid. Specific bands of approximately 168 bp were visualised for all replicates of recombinant plasmid dilutions, except the concentration of 1 × 10^−1^ copies/*μ*L. For recombinant plasmid dilution with a concentration of 1 × 10^−1^ copies/*μ*L only one specific amplicon to three replicates was visualised. MK: MassRuler Low-Range DNA Ladder (Fermentas, Burlington, Ontario, Canada). a, b, c, d, and e: recombinant plasmid dilutions with concentrations of 1 × 10^3^, 1 × 10^2^, 1 × 10^1^, 1 × 10^0^, and 1 × 10^−1^ copies/*μ*L, respectively. W: no template control (water).

The LOD determined on the standard curve generated by amplifying the recombinant plasmid dilutions was found to be 0.0045 fg or 1 copy/*μ*L, thus showing a high sensitivity of the assay. The specificity of the reaction was confirmed by a melting temperature of 83.3°C for standard plasmid dilutions, indicating the formation of a single PCR product with no artefacts, such as nonspecific amplification products or primer dimers (results not shown). Furthermore, amplification products were also checked on agarose gel stained with ethidium bromide in standard TAE buffer and a clear and well-defined specific band of approximately 168 bp was visualised for all replicates of recombinant plasmid dilutions, except the concentration of 1 × 10^−1^ copies/*μ*L. (Figure 3). 

**Figure 4:**
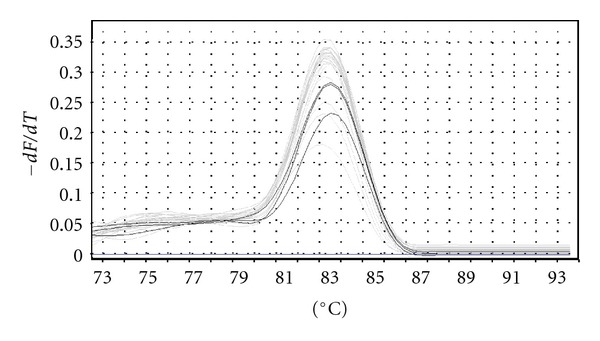
Melting curve analysis of standard plasmid dilutions and sample 893/09-11. In gray: signals obtained from the standard plasmid dilutions; in black: signals obtained from the sample 893.09-11; derivative −*dF*/*dT* where *F* is fluorescence and *T* is time; °C: temperature (centigrade).

The developed assay was capable of detecting bat-SARS-like CoV sample 893/09-11 and no positive results were obtained with any other RNA or DNA viruses tested. The melting curve analysis of sample 893/09-11 showed a single peak at 83.3°C (Figure 4). These results suggested that the technique was highly specific for the bat-SARS-like coronaviruses.

The intraassay variability was determined, first, on seven successive 10-fold dilutions of recombinant plasmid tested in triplicate and, then, on three bat samples with different viral concentrations tested in triplicate. The coefficient of variation (CV) obtained ranged from 0.45 to 1.41 for plasmid concentrations higher than 10^2^ copies/*μ*L, while they increased progressively for lower concentrations. For bat samples, the CV obtained was 1.27 for the viral concentration of 10^2^ copies/*μ*L and 7.56–13.68 for concentrations of 10^0^ copies/*μ*L (Table 2). The determination of the interassay variability tested on three bat samples gave a CV of 1,38 for the viral concentration of 10^2^ copies/*μ*L and 23.97–28.79 for concentrations of 10^0^ copies/*μ*L (Table 2).

The intra- and interassay variability for plasmid dilutions and samples is therefore low for decreasing concentrations down to 10^2^ copies/*μ*L and progressively higher for lower concentrations, as influenced by distribution statistics (Poisson's law) which involves an increase in CV values for the quantification of very low copy number. However, despite the higher variability, low viral concentrations were always detected in all repetitions using the SYBR Green assay.

Furthermore, the standard deviation (SD) between the individual assays was below 0.25 log_10_ (Table 2), a level which is normally considered the minimum requirement for acceptable reproducibility of quantitative molecular assays.

### 3.2. Detection of Coronavirus in Bat Faecal Samples Using SYBR Green Real-Time PCR

Faecal samples of 45 greater horseshoe bats (*Rhinolophus ferrumequinum*) were tested in triplicate for the presence of SARS-like coronaviruses; 19 were positive with a prevalence of 42% (Table 1).

Viral quantities in positive samples ranged from orders of magnitude 10^0^ to 10^2^; a low viral concentration which could also be a consequence of the dilution of the faeces sampled during the extraction procedure. Melting curve analysis showed a single peak between 83° and 84°C for each positive bat sample. The difference in the melting temperature between the standard plasmid (83.3°C, see above) and the faecal samples was a probable consequence of nucleotide mutations in the amplified target sequence (results not shown).

**Figure 5:**
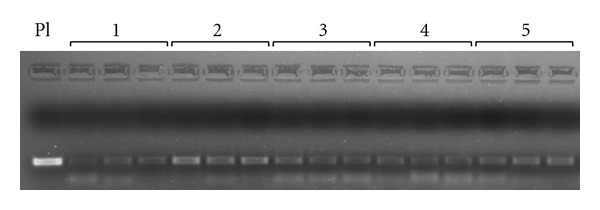
Real time-PCR reaction products checked on agarose gel stained with ethidium bromide in standard tris-acetate-EDTA (TAE) buffer: positive samples detected by developed real-time PCR. Specific bands of approximately 168 bp were visualised for all replicates of the detected positive samples. In this figure is represented the 2% (w/v) agarose gel electrophoresis of five of the 11 positive samples detected in bats belonging to sampling area A (San Cesario sul Panaro, MO). Pl: amplicon of the recombinant plasmid with 1 × 10^4^ copies/*μ*L; 1, 2, 3, 4, and 5: amplicons of the five positive samples belonging to sampling area A, each with three repetitions.

The real-time PCR products were checked on agarose gel stained with ethidium bromide in standard TAE buffer and all replicates of the detected positive samples showed a well-defined specific band of approximately 168 bp (Figure 5).

To confirm that positive samples belonged to SARS-like CoVs, the genome of some detected virus was partially sequenced and analysed by BLAST web interface (http://blast.ncbi.nlm.nih.gov/Blast.cgi) (data not shown).

## 4. Discussion

The importance of bats as a reservoir of infectious viral agents potentially transmissible to humans and other animals has become more evident with the passage of time. Among the different viral families hosted by the bats, much attention has been paid to the coronaviruses which demonstrate considerable variability in these hosts. This is also associated with the central role of Chiroptera as a source of coronaviruses closely related to the SARS virus which, no more than ten years ago, caused a serious global epidemic in humans.

In view of this, there have been numerous studies in recent years with the goal of finding new species of coronaviruses in bats in order to monitor the world situation, to study the possible origin of human SARS virus, and to predict possible new coronavirus outbreaks in humans.

Until now, the predominance of surveys on coronavirus infection in bats has used a reverse-transcribed PCR (RT-PCR) technique for the initial faecal sample screening. However, since conventional PCRs usually have a relatively higher limit of detection in comparison to other methods of analysis, it is possible that there has been an underestimation of the real prevalence of infection in bats.

This possible underestimation of coronavirus prevalence can affect the evaluation of the real epidemiologic impact of bats in viral ecology, by not considering a population of chronically or persistently infected animals with a low viral load, which may be important for the transmission, maintenance, and evolution of the virus in the environment.

In this paper, the description and validation of an SYBR Green real-time PCR method was carried out to detect the presence of SARS-like-coronaviruses, in order to improve the sensitivity of viral detection and to develop, at the same time, a rapid and robust technique to be used for the initial screening of faecal samples.

The SYBR Green real-time PCR developed in this study was highly sensitive, capable of detecting minimum concentrations of virus. Furthermore, it showed optimal efficiency and linearity of the reaction associated with low intra- and interassay variations.

Therefore, this reliable and rapid technique, given its extreme sensitivity and reproducibility, may represent an important and useful alternative method for SARS-like-coronavirus detection in epidemiological investigations. Real-time PCR could be especially useful in programs of bats screening for the detection of viruses from faecal samples having low amounts of viral copies.

The assay developed was applied to the detection of SARS-like coronaviruses in the faeces of bats sampled in different areas of Italy in 2009. Nineteen of the 45 greater horseshoe bats sampled were positive with a 42% prevalence and viral quantities in positive samples ranged from orders of magnitude 10^0^ to 10^2^. Drexler et al. are the only authors that applied a real-time PCR assay to faecal samples of European (Bulgaria) field bats detecting 26% positive animals reaching up to 10^8^ copies per gram of faces [5]. The prevalence of SARS-like coronavirus infection resulted higher in Italian bats than in Bulgarian bats. The Italian bat faeces were initially diluted and not quantified; therefore it is difficult to compare the difference of copies of virus in tested faeces between our and Drexler et al. studies.

The prevalence detected in this survey is much higher than that reported in the only other study done until now regarding the presence of coronavirus in the Italian bat population [4], which detected (using qualitative RT-PCR) two positives out of 52 bats of the same species. These divergent results may reflect a genuine difference in the prevalence of coronavirus infection among bat populations in Italian areas, but it could also be due to the different detection methods used.

## 5. Conclusion

The real-time PCR assay which was developed is a reliable, specific, and sensitive tool with potential utility for rapid screening in bat populations. If conventional qualitative RT-PCR is widely applied (since it allows the sequencing of the obtained products and the genomic characterisation of the viruses), a combined approach with a quantitative real-time PCR furnishes relevant information about the epidemiological situation of coronavirus infection in bats, thus obtaining a more realistic vision of the viral prevalence in the population.

## Figures and Tables

**Table 1:** Bats tested for coronavirus infection using SYBR Green real-time PCR assay.

	Location	Date	No. of bats	Positives
A	San Cesario sul Panaro, MO	16/08/2009	11	10
B	Sigillo, PG	18/09/2009	1	0
C	Monte Croara, S. Lazzaro, BO	05/10/2009	2	0
D	Piobesi d'Alba, CN	11/10/2009	10	0
E	Rossana, CN	11/10/2009	12	3
F	Giovo, SV	19/10/2009	3	3
G	Val di Trebbia, PC	20/10/2009	3	1
H	Castell'arquato, PC	04/11/2009	3	2

	Total		45	19

**Table 2:** Intra- and interassay variability of the SYBR green real-time PCR method.

Intra- and interassay variability				
Samples	Replicate (and assay) numbers	Mean (SD)	log_10_ mean (SD)	log_10_ CV%

Intraassay variability				
A1	3 (1)	1,86*E* + 06 (2,35*E* + 05)	6,27 (0,05)	0,84
A2	3 (1)	2,03*E* + 05 (1,77*E* + 04)	5,31 (0,04)	0,71
A3	3 (1)	2,00*E* + 04 (8,96*E* + 02)	4,30 (0,02)	0,45
A4	3 (1)	2,26*E* + 03 (2,53*E* + 02)	3,35 (0,05)	1,41
A5	3 (1)	2,09*E* + 02 (8,22)	2,32 (0,02)	0,74
A6	3 (1)	1,98*E* + 01 (5,29)	1,28 (0,13)	9,99
A7	3 (1)	2,09*E* + 00 (0,44)	0,31 (0,09)	28,58
B1	3 (1)	5,79*E* + 02 (4,65*E* + 01)	2,76 (0,03)	1,27
B2	3 (1)	2,00*E* + 00 (0,10)	0,30 (0,02)	7,56
B3	3 (1)	4,01*E* + 00 (0,74)	0,60 (0,08)	13,68

Interassay variability				
B1	3 (3)	6,08*E* + 02 (5,58*E* + 01)	2,78 (0,04)	1,38
B2	3 (3)	2,06*E* + 00 (0,34)	0,29 (0,07)	23,97
B3	3 (3)	3,11*E* + 00 (0,82)	0,46 (0,13)	28,79

Successive 10-fold dilutions of recombinant plasmid: A1 (2 × 10^6^), A2 (2 × 10^5^), A3 (2 × 10^4^), A4 (2 × 10^3^), A5 (2 × 10^2^), A6 (2 × 10^1^), and A7 (2 × 10^0^).

Three bat samples with different viral concentrations: B1 (10^2^), B2 and B3 (10^0^).

SD: standard deviation.

CV: coefficient of variation.

## Abbreviations

bat-SARS-like CoV: Bat virus related to SARS-coronavirus
CDV: Canine distemper virus
CN: Copy number
CoV: Coronavirus
Ct: Threshold cycle
CV: Coefficient of variation
−*dF*/*dT*: Derivative where *F* = fluorescence and *T* = time
*E*: Reaction efficiency
FCoV: Feline coronavirus
FPV: Feline panleukopenia virus
IBV: Infectious Bronchitis Virus
LOD: Limit of detection
ORF: Open reading frame
PCR: Polymerase chain reaction
*R*2: Coefficient of determination
RdRp: RNA-dependent RNA polymerase gene
RT-PCR: Reverse transcribed-polymerase chain reaction
*S*: Slope
SARS: Severe acute respiratory syndrome
SARS-CoV: Severe acute respiratory syndrome coronavirus
SARS-like CoV: Severe acute respiratory syndrome-like coronavirus
SD: Standard deviation
TAE: Tris-acetate-EDTA.
